# Thermopile detector of light ellipticity

**DOI:** 10.1038/ncomms12994

**Published:** 2016-10-05

**Authors:** Feng Lu, Jongwon Lee, Aiting Jiang, Seungyong Jung, Mikhail A. Belkin

**Affiliations:** 1 Department of Electrical and Computer Engineering, Microelectronics Research Center, The University of Texas at Austin, Austin, Texas 78758, USA

## Abstract

Polarimetric imaging is widely used in applications from material analysis to biomedical diagnostics, vision and astronomy. The degree of circular polarization, or light ellipticity, is associated with the *S*_3_ Stokes parameter which is defined as the difference in the intensities of the left- and right-circularly polarized components of light. Traditional way of determining this parameter relies on using several external optical elements, such as polarizers and wave plates, along with conventional photodetectors, and performing at least two measurements to distinguish left- and right-circularly polarized light components. Here we theoretically propose and experimentally demonstrate a thermopile photodetector element that provides bipolar voltage output directly proportional to the *S*_3_ Stokes parameter of the incident light.

## Abstract (web summary)

Differences in the intensity of the left- and right-circularly polarized components of light can provide useful information about the chirality of electromagnetic radiation. Here, the authors demonstrate a monolithic photodetector that translates this difference in incident radiation directly into a voltage

## Graphical abstract

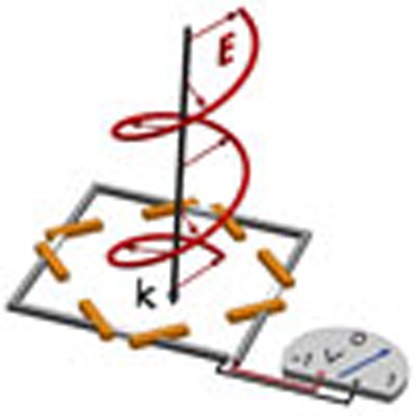

Measurements of light ellipticity or the degree of circular polarization of an electromagnetic wave are highly important for the characterization of chiral molecules^1^,^2^, imaging of biological tissues^3^, identifying bio-organics^4^,^5^, studying cosmic microwave background radiation^6^,^7^, enhancing vision in turbid media^8^,^9^ as well as for performing quantum cryptography and communication experiments^10^,^11^,^12^. Light ellipticity is quantified by the Stokes parameter *S*_3_=*I*_RCP_−*I*_LCP_, where *I*_LCP_ and *I*_RCP_ are the intensities of the left-circularly polarized (LCP) and right-circularly polarized (RCP) light waves in which the electric field moves along a helical trajectory, either clockwise or counterclockwise. Distinguishing the two circular polarizations with conventional photodetectors directly is inherently difficult. Traditionally, external optical components, such as polarizers and wave plates, are used to filter LCP or RCP light for detection^13^,^14^. Intensive investigations have been focused recently on developing integrated solutions for detecting light circular polarization. Detectors based on chiral materials, optical antennas/metamaterials or nonlinear plasmonic structures were proposed and demonstrated with different sensitivity to LCP and RCP light^15^,^16^,^17^,^18^,^19^. However, all these detectors are still sensitive to linearly polarized light, that is, they have non-zero output when *S*_3_=0, and thus cannot be used to measure the *S*_3_ Stokes parameter directly. A number of integrated on-chip photonic elements, such as polarizers^20^,^21^ or beam splitters^22^,^23^,^24^,^25^,^26^,^27^, have also been proposed to separate LCP and RCP light components.

Monolithic detectors with the output directly proportional to the *S*_3_ Stokes parameter would be the most compact and desirable solution for detecting and characterizing light ellipticity, especially if one thinks of polarimetric focal-plane-array imaging, for example, for identification of bio-organics^4^,^5^, astronomical observations^6^,^7^, or vision in turbid media^8^,^9^. Recently, the first monolithic photodetectors with voltage response directly proportional to the light ellipticity were reported in mid-infrared and terahertz spectral ranges^28^,^29^ based on spin-galvanic and circular photogalvanic effect in semiconductors^30^,^31^. However, spin-galvanic effect in semiconductors is intrinsically small^30^,^31^ and thus these detectors require kW cm^−2^-level optical intensity to produce detectable response.

Here we theoretically propose and experimentally demonstrate a thermopile photodetector element with electromagnetically engineered optical antennas that translate the degree of circular polarization of light into the d.c. voltage directly proportional to the *S*_3_ Stokes parameter of the incident radiation. Our detector operation is based on the concept of antenna-coupled thermopiles which are constructed by placing the hot junction of a thermocouple at the centre of an optical antenna and have previously demonstrated sensitivity to linear or mixed light polarization^32^,^33^,^34^,^35^. We demonstrate our detector operation at 7–9 μm wavelength range; however, similar to other thermopile detectors, our detectors can be tailored for operation at any wavelength of interest from visible light to radio frequencies. They provide orders of magnitude higher sensitivity, compared with the photogalvanic photodetectors^28^,^29^ and can be manufactured into focal-plane-arrays. To the best of our knowledge our devices are the only photodetectors, other than that based on photogalvanic effect, that are sensitive exclusively to the degree of circular polarization of light.

## Results

### Thermopile antenna design

**Figure 1:**
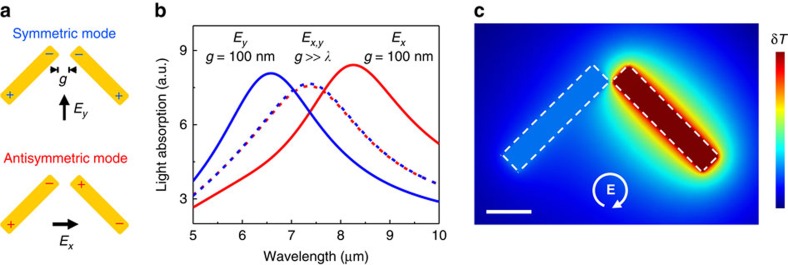
Dimer antenna for circularly polarized light discrimination. (**a**) Basic dimer antenna geometry and associated symmetric (top) and antisymmetric (bottom) plasmon modes. The two rod antennas are positioned orthogonally to each other and are separated by a small gap *g*. (**b**) COMSOL-simulated absorbance of the dimer antenna for **a** for *x*- and *y*-polarized light illumination (red and blue lines, respectively) as a function of the light wavelength *λ*. Solid lines are for *g*=100 nm<<*λ*, dashed lines are for *g*>>*λ*. (**c**) COMSOL simulation of the temperature distribution in the dimer antenna under RCP illumination at the resonant wavelength *λ*=7.5 μm. For simplicity of calculations, the antenna is assumed to be positioned in air and δ*T* is given in arbitrary units. Scale bar, 1 μm. (The temperature distribution in the dimer antenna under LCP illumination is the mirror image of that shown in **c**.)

**Figure 2:**
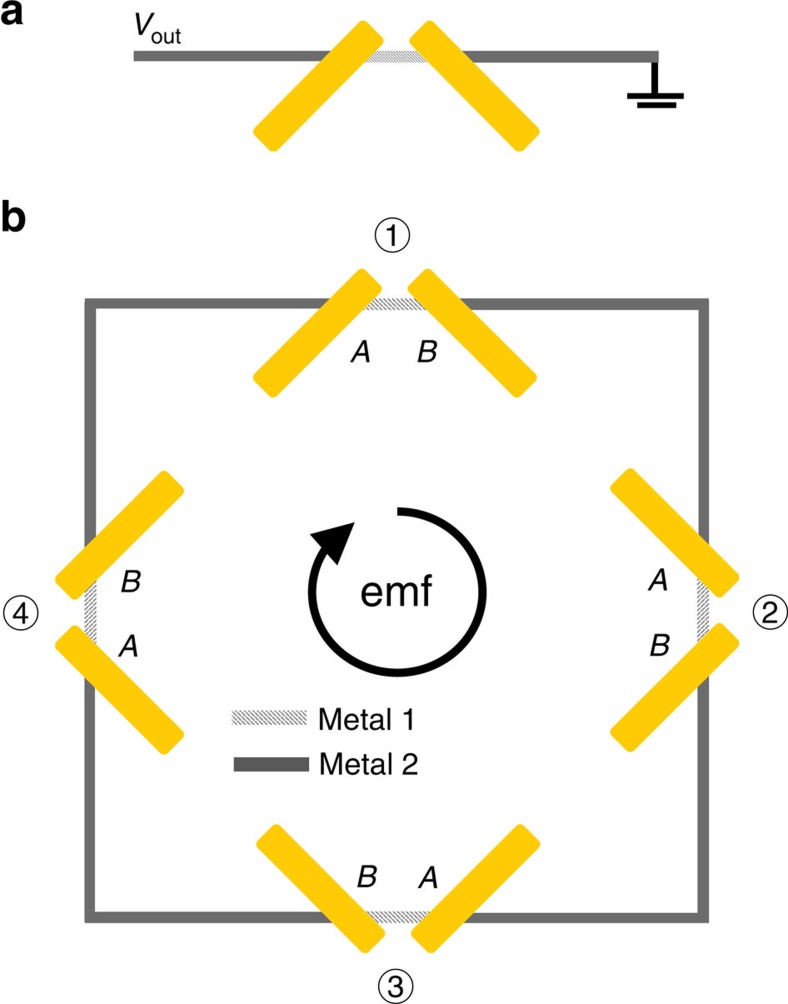
Antenna-coupled thermopile for light ellipticity detection. (**a**) Dimer-antenna-based thermocouple to discriminate between LCP and RCP light. (**b**) The design of the antenna-coupled thermopile element for the detection of *S*_3_ Stokes parameter of light.

Consider two identical rod antennas positioned at ±45° to the *y* axis on a planar substrate as shown in Fig. 1a. The dimensions of the antennas are chosen so that they have a resonance at the target wavelength of 7.5 μm. When the antenna gap is large (*g*>>*λ*), *x*- and *y*-polarized light will excite antisymmetric and symmetric plasmon modes, respectively, as shown in Fig. 1a, with resonances at exactly the same frequencies. However, as the antenna gap decreases to the subwavelength scale, a frequency gap emerges between the resonant positions of the new symmetric and antisymmetric eigenmodes of the dimer antenna^36^. The computed resonance positions of the two modes for the antenna gap of 100 nm are shown in Fig. 1b. Under LCP or RCP illumination, the symmetric and antisymmetric charge oscillations in the dimer antenna will have a relative phase delay given by the sum of the phase delay between *E*_*x*_ and *E*_*y*_ components of the optical field that excite them (±*π*/2 for LCP and RCP light) and the phase delay due to different detuning of symmetric and antisymmetric antenna resonances relative to the excitation light frequency. We adjusted the antenna gap size so that the latter effect produces a phase delay of ∼*π*/2 between symmetric and antisymmetric charge oscillations for the excitation light wavelength of *λ*≈7.5 μm. The total phase difference between the symmetric and antisymmetric charge oscillations induced by circularly polarized light in the dimer antenna is then either 0 or *π* , depending on whether we use LCP or RCP illumination. The induced optical currents of the symmetric and antisymmetric modes then add constructively in one rod antenna in the dimer and destructively in the other. As a result, the LCP (RCP) light produces heating only in the left (right) rod antenna as shown in Fig. 1c. If a thermocouple is now placed in thermal contact with the antennas, as shown schematically in Fig. 2a, the temperature difference between the thermocouple junctions will be translated into the d.c. voltage through Seebeck effect^37^,^38^, which would change sign for LCP and RCP light illumination.

The voltage output of the antenna-coupled thermopile structure shown in Fig. 2a is still sensitive to linearly polarized light (for example, when incident light is polarized along one of the rod antennas). To build a thermopile element sensitive exclusively to the degree of circular polarization of light, we arrange four dimer antennas in a two-dimensional (2D) configuration that possesses *D*_4_ symmetry (that is, the mirror symmetry and the four-fold rotational symmetry), see an example in Fig. 2b. To prove this point, we note that the temperature difference across a thermocouple number *N* (*N*=1–4) in Fig. 2b is given as


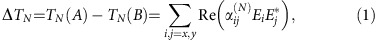


where *T*_*N*_(*A*) and *T*_*N*_(*B*) are the temperatures of the *A* and *B* junctions of the thermocouple *N*, cf. Fig. 2b, *E*_*i*_ and *E*_*j*_ are the components of the normally incident optical field (*i, j*=*x* or *y*) and 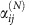 is the complex second-rank tensor relating antenna heating to the optical field components. The values of individual elements in 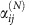 are dependent on the antenna geometry as well as on the thermal and optical properties of the antenna materials, see Supplementary Note 1. The electromotive force (emf) voltage produced by all the four thermocouples in Fig. 2b is given as 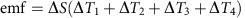, where Δ*S* is the difference in the Seebeck coefficients of the two materials forming the thermocouple^37^,^38^. Using equation (1) and the *D*_4_ symmetry of the antenna structure, we can show the emf voltage is given as


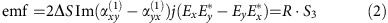


where 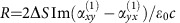 is the detector responsivity (V W^−1^) and 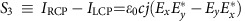 is the *S*_3_ Stokes parameter of the incident light^39^, see Supplementary Note 1 for details of the derivation. The dimer antenna shown in Fig. 1 and discussed above is one of the simplest plasmonic antenna designs with 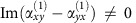. We note that our derivations of equation (2) used only general symmetry considerations so any planar antenna-coupled thermopile with the *D*_4_ symmetry will provide voltage output given by equation (2).

### Experimental implementation

**Figure 3:**
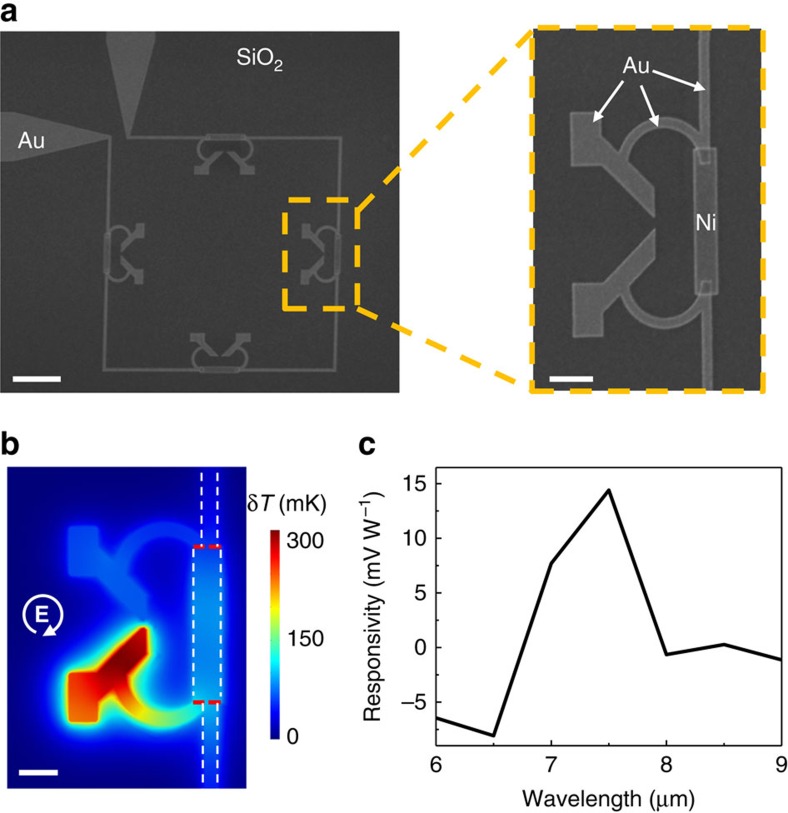
Thermopile element used for experimental measurements. (**a**) Scanning electron microscopy image of the fabricated thermopile element, consisting of V-shaped antennas (made in Au), C-shaped feed lines (Au), thermocouple junctions (Au−Ni) and readout electrodes (top-left corner, Au). Scale bars, 5 and 1 μm (zoom-in). (**b**) COMSOL simulation of the temperature distribution in the antenna structure at the end of a 1-μs-long RCP pulse at *λ*=7.5 μm and 40 W cm^−2^ intensity. The antenna structure is simulated to be on top of a *λ*/4 thick SiO_2_ spacer on the gold substrate. Scale bar, 1 μm. (**c**) Calculated responsivity of the thermopile in **a** for RCP light based on thermal simulations performed at various input light wavelengths. The value was deduced from the simulated temperature at *A* and *B* thermocouple junctions marked by the red lines in **b**.

For the proof-of-concept experimental demonstration, we have fabricated antenna-coupled thermopile element shown in Fig. 3a. Fabrication details are given in the ‘Methods' section. The overall symmetry of the structure in Fig. 3a is similar to that in Fig. 2b and the operating principle is the same. Modifications compared with Fig. 2b are introduced to compensate for distortions in the antenna plasmonic resonances introduced by the presence of thermocouple elements and the substrate. The dimer antennas are connected to the Au–Ni thermocouples by C-shaped gold lines that serve as heat viaducts. Electrodes in the upper-left corner of Fig. 3a are used to read the emf voltage induced in the thermopile loop. Figure 3b shows the simulated temperature map of the antenna in Fig. 3a under 1-μs-long RCP pulse illumination at *λ*=7.5 μm with 40 W cm^−2^ intensity. Given the value of Δ*S*_Au−Ni_=12 μV K^−1^ (ref. 40) and the size of our detector element of 24 × 24 μm^2^, the simulation results translate into the expected thermopile responsivity under under continuous wave (CW) illumination of ∼15 mV W^−1^. The detailed comparison of thermocouple heating dynamics under pulsed and CW illumination is given in Supplementary Fig. 1 and Supplementary Note 2. Figure 3c shows the simulated detector responsivity as a function of light wavelength, which shows a peak at *λ*=7.5 μm. From equation (2) it follows that the spectral responsivity of the detector scales with the wavelength dependence of 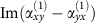. Our dimer antennas were optimized to maximize this parameter at *λ*=7.5 μm and its value drops as the light wavelength is tuned away from antenna resonances.

### Optical characterization

**Figure 4:**
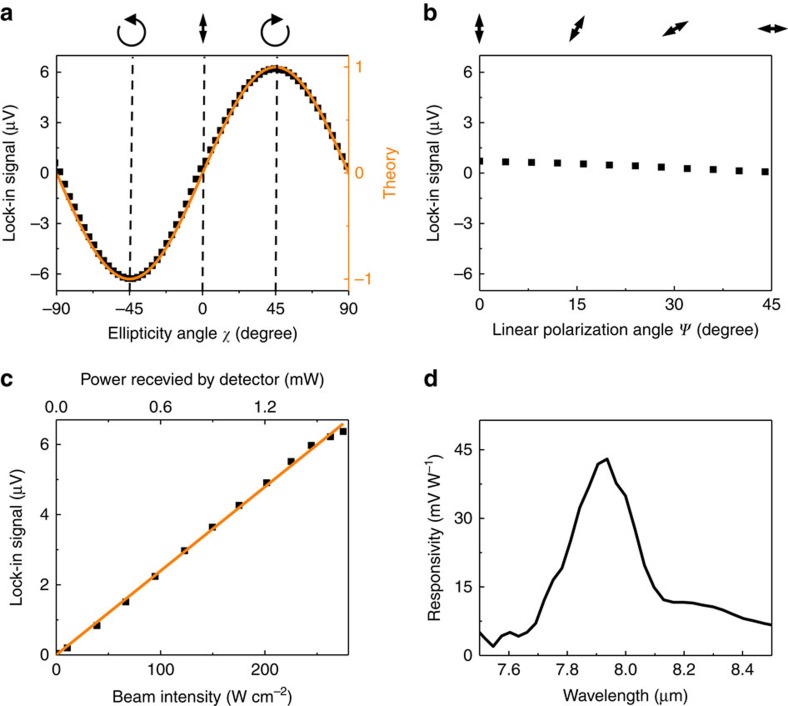
Device characterization. (**a**) Measured thermopile emf voltage (black dots) as a function of the ellipticity angle *χ* of incident light at *λ*=7.9 μm and 270 W cm^−2^ intensity, in comparison with the normalized *S*_3_ Stokes parameter (orange curve). The ellipticity angle *χ* at −45°, 0° and 45° correspond to LCP, linear and RCP light, respectively. (**b**) Measured emf voltage for the linearly polarized light at different polarization directions. Measurements are performed under the same conditions as in **a**. (**c**) The dependence of emf voltage on the light intensity for RCP illumination at *λ*=7.9 μm. (**d**) Measured spectral dependence of the responsivity of the thermopile detector for RCP light illumination.

To characterize the detector performance, infrared pulses with adjustable ellipticity generated by a quantum cascade laser and a quarter-wave plate (QWP) were normally incident onto the thermopile element. Details of the experimental setup are given in the ‘Methods' section. The measurements were performed at *λ*≈7.9 μm, which corresponds to the peak detector responsivity measured experimentally, and with ∼270 W cm^−2^ input light intensity. Figure 4a compares the detector voltage output with the normalized *S*_3_ Stokes parameter of the incident light as a function of the ellipticity angle *χ*, with *χ* defined as sin(2*χ*)=(*I*_RCP_−*I*_LCP_)/(*I*_RCP_+*I*_LCP_)=*S*_3_/*I*_0_ (ref. 39). The bipolar voltage output of the detector is in an excellent agreement with the *S*_3_ Stokes parameter of the incident light, as expected theoretically. We have also tested the detector response to the linearly polarized light at different polarization angles *ψ* (*ψ*=0 corresponds to the light polarization in vertical direction in Fig. 3a). The results of these measurements are shown in Fig. 4b. As expected, our detectors are virtually insensitive to linearly polarized light, as such response is forbidden by symmetry, see equation (2). The extinction ratio of our detector, defined as the ratio of the maximum detector output under linear polarization illumination (that is, the maximum signal in Fig. 4b) to the detector output under purely RCP or LCP illumination, for the same light intensity, is ∼1/10. The residual non-zero response in Fig. 4b may be attributed to (i) non-ideal and non-identical fabrication of the Au–Ni thermocouples in the antenna loop (Fig. 3a), which may lead to different thermal contacts and different Seebeck coefficients^41^ at each thermocouple junction and (ii) distortion of the *D*_4_ symmetry of the detector by the readout electrodes (seen the upper-left corner in Fig. 3a). We note that the second issue may be alleviated by using vertical electrodes or designing similar electrode structures at all four corners of the thermopile antenna structure.

The dependence of the thermopile output on the light intensity is shown in Fig. 4c. As expected, the detector shows linear response to the power of the optical beam. The spectral dependence of the responsivity of the detector is shown in Fig. 4d and is in a good agreement with the theoretical predictions shown in Fig. 3c. The peak detector responsivity is measured to be *R*=43 mV W^−1^ at *λ*≈7.9 μm, which is close to the theoretical expectations presented above. Differences in the peak responsivity value and spectral position are likely the result of differences in the actual values of optical, thermal and thermoelectric parameters of the detector materials, compared with the table values assumed in the simulations, particularly taking into account the effect of nanoscale dimensions of our antenna elements^42^.

## Discussion

We note that we did not aim to produce thermopile detectors with the highest possible sensitivity in this proof-of-concept demonstration. Thermopiles with orders of magnitude higher responsivity, for example, 30 V W^−1^ (ref. 34), may be fabricated by using thermocouples made of semiconductor materials with large difference in their Seebeck coefficients (for example, BiSb/Sb with Δ*S*≈135 μV K^−1^ (refs 32, 34)) and by reducing the heat mass and improving thermal isolation of thermocouple junctions (for example, by using air bridges with conventional materials^32^ or graphene^43^). Air-bridge antenna-coupled BiSb/Sb thermopile detectors have recently demonstrated noise equivalent power below 100 pW Hz^−1/2^ (ref. 34) and we expect that similar levels of sensitivity could be achievable with the detectors presented here. Even in the present form, the sensitivity of our detectors is already orders of magnitude higher than that of detectors based on the circular photogalvanic effect in semiconductors^28^,^29^, which, to the best of our knowledge, are the only other photodetectors that have shown voltage output directly proportional to the degree of circular polarization of the incident light.

To summarize, we proposed the concept and experimentally demonstrated the operation of a novel class of antenna-coupled thermopile photodetectors that provide bipolar voltage response directly proportional to the *S*_3_ Stokes parameter of the incident light. The detector design is completely achiral and the chirality of the incident light is translated into the direction of current and/or the sign of d.c. voltage in the detector. Given the compactness and simplicity of the photodetectors presented here and the CMOS compatibility of the thermopile photodetector technology^41^,^44^, we expect that our elements can be easily integrated into various polarimetry systems and be used to provide video-rate focal-plane-array imaging of the *S*_3_ Stokes parameter of light for identification of bio-organics^4^,^5^, astronomical observations^6^,^7^ or vision in turbid media^8^,^9^.

## Methods

### Device fabrication

The device fabrication started from depositing a 100-nm-thick Au film (via e-beam evaporation) on the Si substrate to serve as the bottom reflector, followed by the deposition of a 2-μm-thick layer of SiO_2_ (via plasma-enhanced chemical vapor deposition (PECVD)) on top of the Au film. The SiO_2_ layer serves as the *λ*/4 dielectric spacer which ensures that the wave reflected from the ground plane interferes constructively with the incident wave on the antenna surface. Au was chosen as the material for the optical antenna as well as part of the thermocouple, and was joined by Ni to form the thermocouple junction. The antenna-coupled thermopile structure was fabricated by the e-beam lithography, metal deposition and lift-off. The structure elements are made with 50-nm-thick Au antenna/thermocouple, 60-nm-thick Ni thermocouple and lastly 100-nm-thick Au electrodes. Ti (5–10 nm thick) was deposited before any Au layer to promote adhesion. The active region of the device had a footprint of 24 × 24 μm^2^. After fabrication, the device was wire bonded to the chip carrier for testing with electrostatic discharge precautions.

### Experimental setup

A tunable quantum cascade laser (Daylight Solutions) was used as the mid-infrared source. The output was 1-μs-long pulses repeated at the rate of 100 kHz. The quantum cascade laser beam was inherently linearly polarized, and was converted to be elliptical by passing through a QWP (Altechna). In this configuration, the ellipticity angle *χ* equals the angle *θ* between the optical axis of QWP and light linear polarization direction 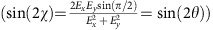, which can be continuously tuned. The beam was then focused onto the thermopile detector at normal incidence using a ZnSe lens with 6 inch focal length, resulting in a beam spot size of ∼500 μm in diameter. The thermopile emf voltage was recorded using the lock-in amplifier (Stanford Research SR830) referenced by the laser pulse trigger. Details of extracting the value of the thermopile voltage from the lock-in amplifier output are given in Supplementary Note 2. To measure the device response to linearly polarized light, QWP was replaced by a half-wave plate (Altechna) and a wire-grid polarizer.

### Data availability

The data that support the findings of this study are available from the corresponding author upon request.

## Additional information

**How to cite this article:** Lu, F. *et al*. Thermopile detector of light ellipticity. *Nat. Commun.* **7,** 12994 doi: 10.1038/ncomms12994 (2016).

## Supplementary Material

Supplementary Information Supplementary Figure 1 and Supplementary Notes 1-2
