# Supplementary material for: Thermopile detector of light ellipticity
Source: Nat Commun. 2016 Oct 5;7:12994. doi: 10.1038/ncomms12994 (PMC5059469; doi:10.1038/ncomms12994)
Supplement: Supplementary Information — Supplementary Figure 1 and Supplementary Notes 1-2 [file ncomms12994-s1.pdf]

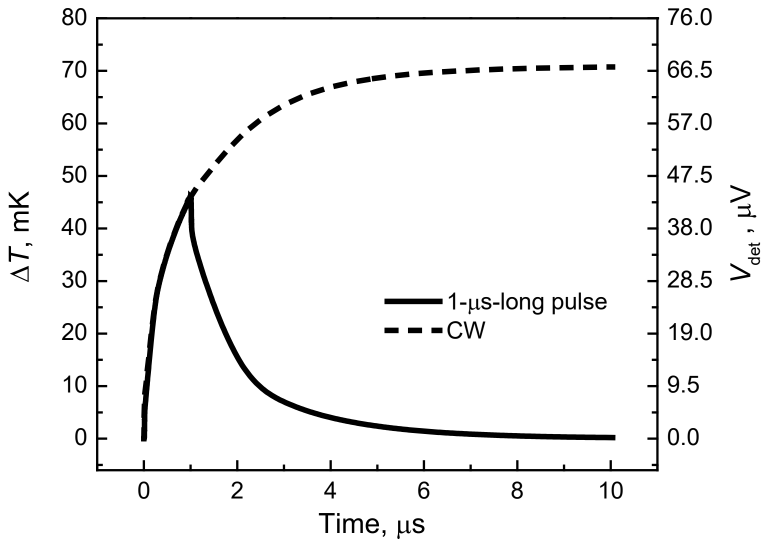

Supplementary Figure 1. COMSOL simulation of the time-dependent temperature difference ΔT between the two thermocouple junctions under pulsed (solid curve) and CW (dashed curve) circularly polarized light illumination at the resonant wavelength with 40 W cm^{-2} light intensity. The right axis corresponds to the instantaneous detector voltage V_{det} under the experimental condition (270 W cm^{-2} light intensity), which was deduced from the lock-in output using the method discussed in Supplementary Note 2.

Supplementary Note 1

Equations 1 and 2 used in the main text are derived below.

The Joule heating power P at any point $\mathbf{r}$ of the thermopile structure depicted in Fig. 2b is given as

$$P(\mathbf{r}) = \frac{1}{2}\mathrm{Re}\left(\mathbf{J}(\mathbf{r})\cdot\mathbf{E}^{*}_{\mathrm{loc}}(\mathbf{r})\right), \tag{S1}$$

where $\mathbf{J}(\mathbf{r})$ is the local 2D current density and $\mathbf{E}^{*}_{\mathrm{loc}}(\mathbf{r})$ is the complex conjugate of the local electric field, both oscillating at the input light frequency. Assuming linear response in our system, both $\mathbf{J}(\mathbf{r})$ and $\mathbf{E}^{*}_{\mathrm{loc}}(\mathbf{r})$ must be linearly proportional to the input field amplitude components E_x and E_y. As a result, Eq. (S1) can be rewritten as

$$P(\mathbf{r}) = \frac{1}{2}\mathrm{Re}\left(\sigma_{ij}(\mathbf{r})E_iE_j^{*}\right), \tag{S2}$$

where $\sigma_{ij}(\mathbf{r})$ is a 2-dimensional matrix of complex coefficients that may be position-dependent, and E_i and E_j are the components of the input optical field ($i, j = x$ or y), and the summation over the repeated indices is implied.

The temperature increase over the substrate temperature at thermoelectric junction A of thermocouple 1, $\delta T_1(A)$ is produced by integrating contribution of the Joule heating from the entire thermopile antenna structure (taking into account thermal transport). The overall result depends on the exact antenna geometry as well as on the thermal properties of the materials composing the detector. However, from Eq. (S2), the result will still be linearly proportional to $E_iE_j^{*}$:

$$\delta T_1(A) = \mathrm{Re}\left(\alpha_{ij}^{(1A)}E_iE_j^{*}\right), \tag{S3}$$

where $\alpha_{ij}^{(1A)}$ is the matrix of complex coefficients, $i, j = x$ or y, and the summation over repeated indices is implied.

Similarly, the temperature increase over the substrate temperature at thermoelectric junction B of thermocouple 1, $\delta T_1(B)$ is given as:

$$\delta T_1\left(B\right) = \mathrm{Re}\left(\alpha_{ij}^{(1B)} E_i E_j^*\right), \tag{S4}$$

where $\alpha_{ij}^{(1B)}$ is the matrix of complex coefficients, $i, j = x$ or y, and the summation over repeated indices is implied. From the mirror symmetry of the thermocouple it follows that $\alpha_{xx}^{(1A)} = \alpha_{xx}^{(1B)}$ and $\alpha_{yy}^{(1A)} = \alpha_{yy}^{(1B)}$.

The temperature difference between thermocouple 1 junctions is given as

$$\Delta T_1 = \delta T_1\left(A\right) - \delta T_1\left(B\right) = \mathrm{Re}\left(\alpha_{ij}^{(1)} E_i E_j^*\right), \tag{S5}$$

where $\alpha_{ij}^{(1)} = \alpha_{ij}^{(1A)} - \alpha_{ij}^{(1B)}$. Since $\alpha_{xx}^{(1A)} = \alpha_{xx}^{(1B)}$ and $\alpha_{yy}^{(1A)} = \alpha_{yy}^{(1B)}$, we have $\alpha_{xx}^{(1)} = \alpha_{yy}^{(1)} = 0$. From the C_4 symmetry of the entire antenna structure, it follows that $\alpha_{xy}^{(1)} = -\alpha_{yx}^{(2)} = \alpha_{xy}^{(3)} = -\alpha_{yx}^{(4)}$, and $\alpha_{yx}^{(1)} = -\alpha_{xy}^{(2)} = \alpha_{yx}^{(3)} = -\alpha_{xy}^{(4)}$. The *emf* voltage produced by the thermocouples in such an antenna is then given as

$$\begin{gathered} emf = \Delta S\left(\Delta T_1 + \Delta T_2 + \Delta T_3 + \Delta T_4\right) = \\ \Delta S\,\mathrm{Re}\left(\alpha_{xy}^{(1)} E_x E_y^* + \alpha_{yx}^{(1)} E_y E_x^*\right) + \Delta S\,\mathrm{Re}\left(\alpha_{xy}^{(2)} E_x E_y^* + \alpha_{yx}^{(2)} E_y E_x^*\right) + \\ \Delta S\,\mathrm{Re}\left(\alpha_{xy}^{(3)} E_x E_y^* + \alpha_{yx}^{(3)} E_y E_x^*\right) + \Delta S\,\mathrm{Re}\left(\alpha_{xy}^{(4)} E_x E_y^* + \alpha_{yx}^{(4)} E_y E_x^*\right) = \\ \Delta S\,\mathrm{Re}\left(\alpha_{xy}^{(1)} E_x E_y^* + \alpha_{yx}^{(1)} E_y E_x^*\right) + \Delta S\,\mathrm{Re}\left(-\alpha_{yx}^{(1)} E_x E_y^* - \alpha_{xy}^{(1)} E_y E_x^*\right) + \\ \Delta S\,\mathrm{Re}\left(\alpha_{xy}^{(1)} E_x E_y^* + \alpha_{yx}^{(1)} E_y E_x^*\right) + \Delta S\,\mathrm{Re}\left(-\alpha_{yx}^{(1)} E_x E_y^* - \alpha_{xy}^{(1)} E_y E_x^*\right) = \\ 2\Delta S\,\mathrm{Im}\left(\alpha_{xy}^{(1)} - \alpha_{yx}^{(1)}\right) j\left(E_x E_y^* - E_y E_x^*\right), \end{gathered} \tag{S6}$$

which is the same as Eq. (2) in the main text.

Supplementary Note 2

To deduce the detector's peak responsivity, we simulated the heating and cooling dynamics of the thermocouple under one 1-μs-long pulse illumination (repeated at 100 kHz). The result is plotted as the solid curve in Supplementary Figure 1 (left axis), where ΔT is the temperature difference between the two thermopile junctions (cf. Fig. 3 in the main text). The dashed curve in Supplementary Figure 1 shows the heating of the thermocouple under CW illumination, in which case the rise time of the detector (10% to 90%) is ~3μs. The output voltage of the lock-in amplifier used in our experiments (V_{out}) is given as:

$$V_{\mathrm{out}} = \frac{\sqrt{2}}{T}\int_0^T V_{\mathrm{det}}(t)\sin\left(2\pi f_{\mathrm{ref}} + \phi\right)dt\,, \tag{S7}$$

where f_{ref} is the reference frequency, $V_{det}(t)$ is the detector voltage as a function of time, and ϕ is the adjustable phase, which was fixed to maximize $|V_{out}|$. The detector $V_{det}(t)$ is expected to follow $\Delta T(t)$ in Supplementary Figure 1. The maximum lock-in output in our experiment was approximately 6 μV for illumination with 1-μs-long square QCL pulses with 270 W cm^{-2} peak intensity at 100 kHz repetition frequency (see Fig. 4). We can then use Eq. (S7) and the time-dependence of ΔT in Supplementary Figure 1 to deduce $V_{det}(t)$ for our thermopile, which is shown in Supplementary Figure 1, right axis. The peak intensity of 270 W cm^{-2} corresponds to peak power of 1.55 mW incident on the 24 × 24 μm^2 detector element, which translates into detector responsivity under CW illumination of approximately 43 mV W^{-1}.
